# Age-dependent impairment of disease tolerance is associated with a robust transcriptional response following RNA virus infection in *Drosophila*

**DOI:** 10.1093/g3journal/jkab116

**Published:** 2021-04-09

**Authors:** Lakbira Sheffield, Noah Sciambra, Alysa Evans, Eli Hagedorn, Casey Goltz, Megan Delfeld, Haley Kuhns, Janna L Fierst, Stanislava Chtarbanova

**Affiliations:** Department of Biological Sciences, University of Alabama, Tuscaloosa, AL 35487, USA

**Keywords:** aging, infection, disease tolerance, RNA virus, Drosophila melanogaster, transcriptomics

## Abstract

Advanced age in humans is associated with greater susceptibility to and higher mortality rates from infections, including infections with some RNA viruses. The underlying innate immune mechanisms, which represent the first line of defense against pathogens, remain incompletely understood. *Drosophila melanogaster* is able to mount potent and evolutionarily conserved innate immune defenses against a variety of microorganisms including viruses and serves as an excellent model organism for studying host–pathogen interactions. With its relatively short lifespan, *Drosophila* also is an organism of choice for aging studies. Despite numerous advantages that this model offers, *Drosophila* has not been used to its full potential to investigate the response of the aged host to viral infection. Here, we show that, in comparison to younger flies, aged *Drosophila* succumb more rapidly to infection with the RNA-containing Flock House virus due to an age-dependent defect in disease tolerance. Relative to younger individuals, we find that older *Drosophila* mount transcriptional responses characterized by differential regulation of more genes and genes regulated to a greater extent. We show that loss of disease tolerance to Flock House virus with age associates with a stronger regulation of genes involved in apoptosis, some genes of the *Drosophila* immune deficiency NF-kB pathway, and genes whose products function in mitochondria and mitochondrial respiration. Our work shows that *Drosophila* can serve as a model to investigate host–virus interactions during aging and furthermore sets the stage for future analysis of the age-dependent mechanisms that govern survival and control of virus infections at older age.

## Introduction

Infectious diseases, including viral infections, represent an important burden among the elderly. For instance, older age is a major risk factor for increased morbidity and mortality to numerous viral pathogens including Influenza virus (flu), West Nile virus, and severe acute respiratory syndrome (SARS) associated coronavirus-2 (SARS-CoV-2), the agent responsible for the current COVID-19 pandemics (Hernandez-Vargas *et al.* 2014; Montgomery 2017; Nikolich-Zugich *et al.* 2020). Immunosenescence, a collective term used to describe the progressive functional decline of the immune system over time, is associated with the increased susceptibility to infections and lower responsiveness to vaccination observed in the elderly (Leng and Goldstein 2010). Considerable progress has been made in understanding how aging affects both, the innate and adaptive immune systems, however, the causes underlying immunosenescence remain incompletely elucidated. In particular, the age-dependent mechanisms leading to dysregulated innate immunity, which represents the first line of defense against invading pathogens, are less well documented (reviewed in Nikolich-Zugich 2018). Moreover, the exact factors and molecular events contributing to the more rapid death of the aged organism following virus infection are not fully understood (Nikolich-Zugich *et al.* 2020). With an increasing aging population (He *et al.* 2016), it remains of primary importance to further our knowledge of the mechanisms underlying the capability of the aged organism to survive infection, and consequently to ensure appropriate preventive and treatment strategies that will improve health in the elderly.

Pioneering research using the genetically tractable model organism *Drosophila melanogaster*, which in contrast to vertebrates is devoid of a classic adaptive immune system, has uncovered conserved mechanisms of activation of innate immunity in response to bacterial and fungal pathogens. Following bacterial or fungal infection, two nuclear factor kappa B (NF-κB) pathways, Toll and immune deficiency (IMD), which share similarities with mammalian Toll-like receptor/interleukin (IL)-1 receptor and tumor necrosis factor receptor (TNFR) pathways, respectively, are activated. These pathways mediate the transcription of downstream effector targets including antimicrobial peptides (AMPs) and immune-induced molecules (IMs) (Lemaitre and Hoffmann 2007; Clemmons *et al.* 2015). *Drosophila* also detect and respond to viral pathogens via multiple mechanisms that mediate antiviral defenses. RNA interference (RNAi), which relies on production of virus-derived small interfering RNAs (siRNAs), provides broad protection against RNA and DNA viruses. Cellular processes such as apoptosis, apoptotic bodies’ clearance by plasmatocytes (macrophage-like cells in *Drosophila*), and autophagy also represent effective antiviral mechanisms (reviewed in Mussabekova *et al.* 2017 and in Lamiable and Imler 2014). In *Drosophila*, viral infections are also associated with complex transcriptional responses that reflect the regulation of cellular pathways, production of cytokines and effector molecules, changes in stress response and physiology (reviewed in Mussabekova *et al.* 2017). Although the *Drosophila* genome does not encode for interferon genes, the protein encoded by the stimulator of interferon genes (STING), which in mammals activates NF-κB and interferon signaling in response to viral infection, is present in this organism. dSTING recently was shown to contribute to antiviral immunity by interacting with some of the components of the *Drosophila* IMD pathway in response to picorna-like viruses (Goto *et al.* 2018) and by activating downstream autophagy in response to ZIKA virus infection in the brain (Liu *et al.* 2018). In addition to these mechanisms that are in control of pathogen burden (also referred as resistance mechanisms), the outcome of infection is determined by the ability of the host to endure the damaging effects caused by the pathogen or resulting from immunopathology (a phenomenon known as disease tolerance). Both resistance and tolerance are considered components of host immunity and effective tolerance mechanisms allow resistance mechanisms to operate in a more optimal way (reviewed in Ayres and Schneider 2012; Martins *et al.* 2019).

Aging in *Drosophila* also leads to deregulation of innate immunity. For instance, expression of several genes encoding AMPs downstream of NF-κB pathways increases with age (reviewed in Garschall and Flatt 2018), similar to inflammaging, the low-grade chronic inflammation that accompanies aging (Franceschi *et al.* 2000) in mammals. Additionally, the phagocytic capacity of *Drosophila* macrophages declines with age (Mackenzie *et al.* 2011; Horn *et al.* 2014). Aged *Drosophila* also are more sensitive to infections with Gram-negative bacteria, Gram-positive bacteria, fungi, and viruses such as *Drosophila* C virus (DCV) and the Flock House virus (FHV) (Ramsden *et al.* 2008; Eleftherianos *et al.* 2011; Fabian *et al.* 2018). However, there is still a very limited understanding of how antiviral immunity operates as a function of age in *Drosophila*. With increasing evidence for impaired defenses against viruses in the aged organism, flies can serve as a prime genetic model of aged host–virus interactions and can offer unique opportunities for mechanistic dissection of age-dependent innate immune responses.

In the present study, we conducted comparative analysis of survival, virus load, and gene expression between young and aged *Drosophila* following infection with the FHV. FHV is a small, non-enveloped insect virus, whose bipartite genome is composed of two positive, single-stranded RNA molecules (Venter and Schneemann 2008). Originally isolated from a grass grub (Dearing *et al.* 1980; Scotti *et al.* 1983), FHV has a broad range of hosts, including *Drosophila*. In *Drosophila*, this virus effectively replicates in the fat body (equivalent of mammalian liver), ovarian egg chamber, the cardiac and muscle tissues, and in trachea, causing lethality (Galiana-Arnoux *et al.* 2006; Eleftherianos *et al.* 2011; Thomson *et al.* 2012; Xu and Cherry 2014). Upon infection, FHV’s genome is delivered into the host cell as messenger RNA (mRNA), ready to be translated, and then amplified by the virally encoded RNA-dependent RNA polymerase (vRdRP). During replication, a subgenomic RNA3 (sgRNA3) is amplified from RNA1, leading to the synthesis of two proteins, including the suppressor of antiviral RNA interference B2. Capsids made of protein alpha (α) assemble with newly synthesized RNA (+) to produce new viral particles that will be released from the cell (Venter and Schneemann 2008). FHV replication both in insect cell culture and *in vivo* is associated with the formation of replication complexes on the outer mitochondrial membrane inducing characteristic spherule-like membrane invaginations (Kopek *et al.* 2007; Eleftherianos *et al.* 2011). Protective resistance mechanisms against systemic FHV infection in *Drosophila* include antiviral RNAi (Galiana-Arnoux *et al.* 2006), the rapid induction of apoptosis (Liu *et al.* 2013), as well as hemocyte-mediated phagocytosis (Lamiable *et al.* 2016). The outcome of FHV infection also involves disease tolerance mechanisms mediated by the Histone H3 lysine 9 (H3K9) methyltransferease G9-alpha (Merkling *et al.* 2015a). FHV infection also leads to complex changes of the *Drosophila* transcriptome. Previous analysis of differential gene expression showed that FHV induces more genes than it represses (Kemp *et al.* 2013; Chtarbanova *et al.* 2014). Among FHV-induced genes, several encode for heat shock proteins (HSPs), glutathione transferases, cytochrome P450s, members of the Turandot (Tot) and thioester-containing protein families, cytoskeletal regulators as well as genes involved in the processes of cell death, phagocytosis, and oxidation–reduction (Kemp *et al.* 2013). FHV downregulated genes include some encoding for AMPs such as *Cecropin* and *Drosocin* as well as accessory gland protein (Acp) genes such as *Acp26Aa*, *Acp62F*, and *Acp63F* (Chtarbanova et al. 2014).

Here, we report that older flies succumb faster to FHV infection without accumulating higher virus loads, suggesting that a tolerance mechanism becomes impaired with age. Additionally, we show that early in the infection process, aged flies mount a more robust transcriptional response to FHV than young flies. This response is associated with the at least twofold regulation of more genes and genes regulated to a greater extent. Differential gene expression analysis also shows that the response of aged flies to FHV differs from the response of flies undergoing aging in absence of infection. This includes different expression profiles for several genes belonging to the “Innate immune response” gene ontology (GO) category. Moreover, we found upregulation to a greater extent of genes encoding for multiple components of the “apoptotic process” GO category in aged, FHV-infected flies. Additionally, we show that several genes whose gene products function in mitochondria and mitochondrial respiratory chain are downregulated in aged, FHV-infected flies in comparison to young flies. We also demonstrate that among genes that do not belong to specific gene ontology categories, the expression of several non-coding RNAs (ncRNAs) changes in aged, FHV-infected flies in comparison to young, FHV-infected *Drosophila* and flies undergoing aging. Collectively, our work shows that virus infection in aged flies triggers profound changes in transcriptomics and establishes *Drosophila* as a model that allows investigation of the age-dependent mechanisms underlying the response and survival to viral infection.

## Materials and methods

### *Drosophila* handling

All *Drosophila* stocks were raised and maintained on Nutri-Fly^®^ Bloomington formulation food (Genesee Scientific, Cat No.: 66-113) at 25°C. *Oregon-R* (No. 2376) and *y^1^ w ^67c23^* (No. 6599) flies were obtained from the Bloomington *Drosophila* Stock Center (Bloomington, IN). *w^1118^* flies were a kind gift from Dr John Yoder (University of Alabama). For aging experiments, 0- to 4-day-old animals were collected, CO_2_-anesthetized, separated by sex, and placed in a 25°C incubator with controlled 12/12 dark/light cycle. Flies were flipped every 2–3 days in a fresh food-containing vial until desired age was reached. For survival and virus load determination young flies were 3- to 7-day old (labeled as 5d-old), and aged flies were 27- to 31-day old (labeled as 30d-old). For RNA-Seq experiments, replicates containing young flies were 6- to 9-day old (labeled as 7d-old), and aged flies were 22- to 29-day old (labeled as 25d-old), Supplementary Table S1. *Wolbachia*-free, non-virgin flies were used in all experiments.

### Virus stock and infections

Flock House virus was a kind gift from Dr Annette Schneemann (Scripps Research Institute, La Jolla, CA). FHV stock titer was determined at 2.92E + 06 TCID50/mL using the method as in (Eleftherianos *et al.* 2011). UV virus treatment (24,000 mJ of UV light) was done as described by (Merkling *et al.* 2015b) using a GS Gene linker UV Chamber (Bio-Rad) (Supplementary Figure S1). Flies of desired sex, age, and genotype were individually injected with 4.6 nL of either virus stock solution or control 10 mM Tris–HCl pH 7.5 solution under CO_2_ anesthesia using a Nanoject II injector (Drummond Scientific). Flies were let to recover from the injection for ∼1 h at room temperature and then were placed in a 22°C incubator. For survival experiments, flies were separated by sex and placed in groups of 10 per vial for each experimental treatment. The number of living flies was recorded every 24 h. For virus load determination by RT–qPCR, flies were separated by sex and frozen in groups of five flies per experimental treatment at 4-, 5-, 6-, and 7-day post-infection (dpi) prior RNA extraction.

### RNA sequencing

The Quick-RNA MiniPrep Kit (Zymo Research) was used to isolate total RNA from 15 whole flies. Three biological replicates were collected for each experimental condition. RNA was extracted following manufacturer’s instructions and sent to Novogene Co., Ltd. for RNA sequencing. Prior directional library preparation, quality of RNA for all samples was evaluated by Novogene Co., Ltd. for purity, degradation, potential contamination, and integrity. Only for samples that passed quality control, mRNA was enriched using oligo(dT) beads. Constructed libraries were quality checked and paired-end sequencing performed using Illumina technology. Bioinformatics analysis to determine differential gene expression was performed by Novogene Co., Ltd using the *Drosophila melanogaster* reference genome (dmel_r6.23_FB2018_04). This data was used in Figures2, 3, 5, and 6, and in Supplementary Figures S5, S6, S10, and S11. Read counts were normalized using the DESeq 1.10.1 (Anders and Huber 2010) method and adjusted *P*-values (*P adj*) estimated based on a negative binomial distribution model. *P adj* <0.05 were considered significant. Read counts were normalized with a regularized log transformation (rlog) for visualization. Validation of gene expression by RT–qPCR was performed on RNA used for the RNAseq experiment. Determination of differential gene expression in experimental groups is as follows: Aging: Non-infected 25d/Non-infected 7d; Young Tris24h: 7d Tris 24h/7d Ni; Young Tris48h: 7d Tris 48h/7d Ni, Aged Tris24h: 25d Tris 24h/25d Ni; Aged Tris48h: 25d Tris 48h/25d Ni; Young FHV24h: 7d FHV 24h/7d Tris 24h; Aged FHV24h: 25d FHV 24h/25d Tris 24h. Young FHV48h: 7d FHV 48h/7d Tris 48h; Aged FHV48h: 25d FHV 48h/25d Tris 48h.

**Figure 1:**
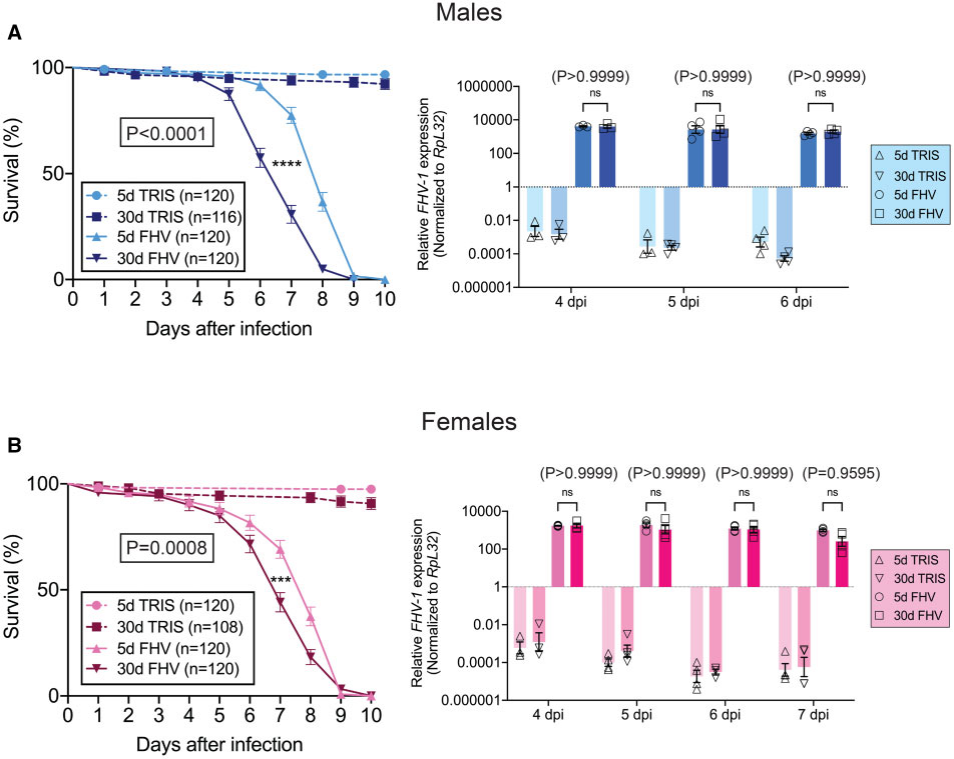
FHV infection triggers more rapid death of aged *Drosophila* without accumulation of higher virus load. (A) Left panel. Survival curves of young and aged male *Oregon R* *Drosophila* that have been infected with FHV or control-injected with the same volume of Tris. The graph compares the survival curves of 5d-old and 30d-old males from 12 independent injection experiments done in groups of 8–10 flies. Right panel. Virus load determined by *FHV RNA1* expression at 4, 5, and 6 dpi reveals comparable titers between young and aged males. Graphs represent mean ± SEM from three to four independent experiments, each done for groups of five flies. (B) Left panel. Survival curves of young and aged female *Oregon R* *Drosophila* that have been infected with FHV or control-injected with the same volume of Tris. The graph compares the survival curves of 5d-old and 30d-old females from 12 independent injection experiments done in groups of 8–10 flies. Right panel. Virus load determined by *FHV RNA1* expression at 4, 5, 6, and 7 dpi reveals comparable titers between young and aged females. Graphs represent mean ± SEM from three to four independent experiments, each done for groups of five flies. (A and B) Statistics of FHV survival are based on a Log-Rank (Mantel-Cox) test. ****P < 0.0001, ***P < 0.001. Specific P-values are indicated in each graph. Statistics for virus load are based on two-way ANOVA followed by Tukey post-test to correct for multiple comparisons. ****P < 0.0001, **P < 0.001, *P < 0.05, ns = non-significant (P > 0.05). Specific P-values are indicated for each time point in each graph.

**Figure 2:**
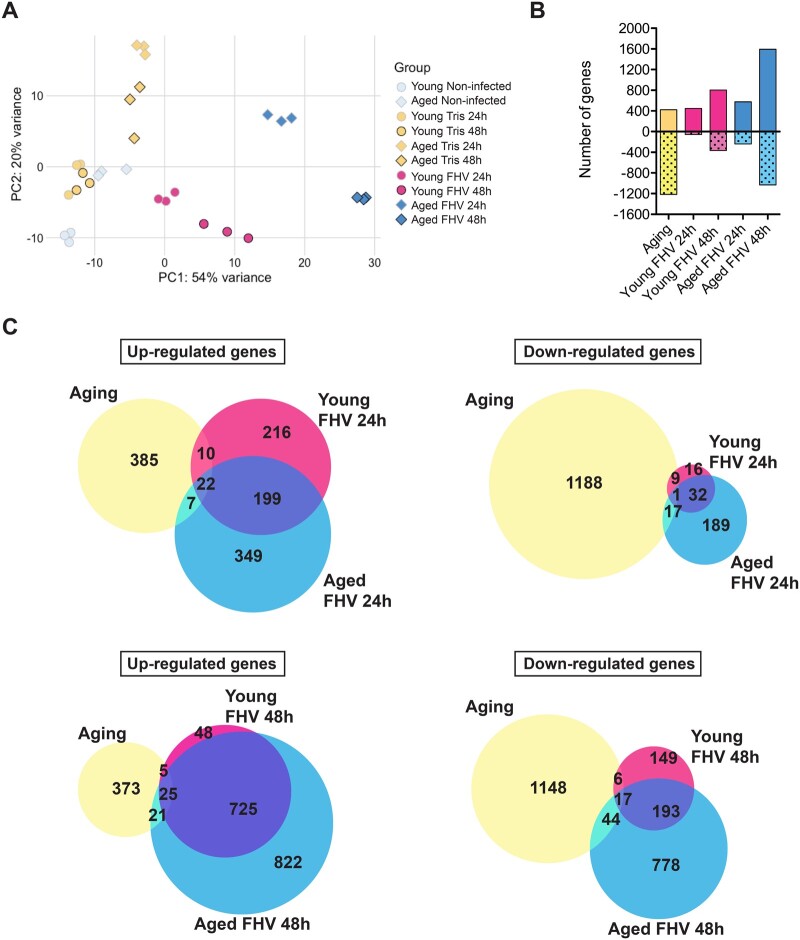
FHV infection of aged flies leads to a robust transcriptional response. (A) Principal component analysis (PCA) for all experimental samples. PCA showing that the majority of the variance observed in the transcriptional response is due to infection treatment (PC1) and age (PC2). (B) Comparison of the number of differentially regulated genes at least twofold in all conditions. Positive values represent upregulated genes and negative values represent downregulated genes. (C) Venn diagrams showing overlaps between differentially regulated genes for selected experimental conditions.

**Figure 3:**
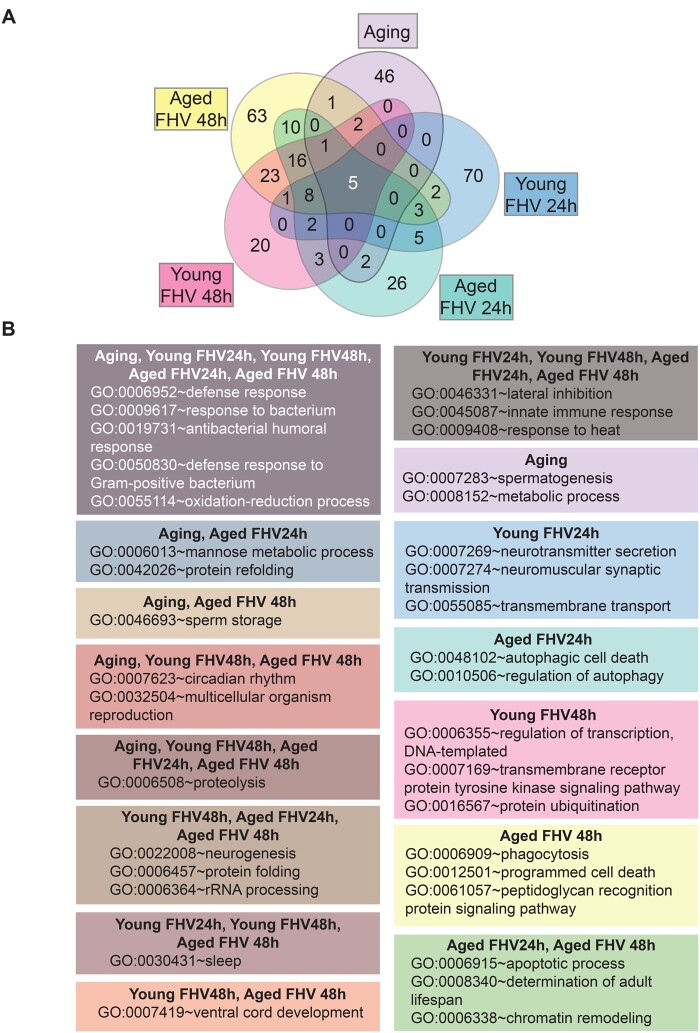
Common and distinct biological processes are regulated by aging and FHV infection in *Drosophila*. (A) Venn diagram showing overlaps between the number of Biological processes among different experimental groups, based on Gene ontology analysis. (B) Selected specific and overlapping GO categories belonging to experimental groups discussed in the text. Complete list of common and overlapping biological processes could be found in Supplementary Table S8.

**Figure 4:**
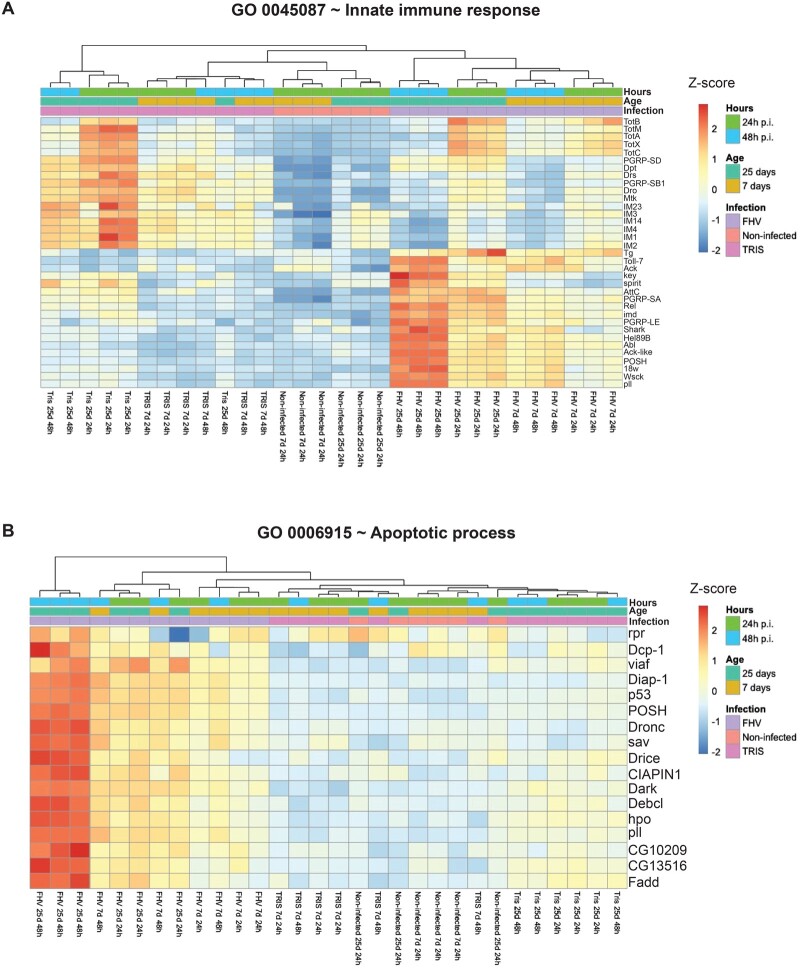
Regulation of innate immunity and programmed cell death genes by aging, Tris-injection, and FHV infection. (A) Heatmap comparing the expression of genes belonging to the GO category “innate immune response” based on their expression in all experimental groups. (B) Heatmap comparing the expression of genes belonging to the GO category “apoptotic process” based on their expression in all experimental groups. (A and B) The expression scale represents the Z-score [Z score = (x−µ)/σ), where x is value, µ is mean, and σ is SD].

### RT–qPCR gene expression analysis

The Quick-RNA MiniPrep Kit (Zymo Research) was used to isolate total RNA following manufacturer’s instructions. RNA (250, 500, or 1000 ng) was converted to cDNA using the High Capacity RNA-to-cDNA Kit (Applied Biosystems). Based on initial RNA quantity used for the reaction, obtained cDNA was diluted 5, 10, or 20 times, respectively and RT–qPCR reaction carried out using *Power* SYBR™ Green PCR Master Mix (Applied Biosystems) according to manufacturer’s instructions. Primer sequences are listed in Supplementary Table S2. For all assays, normalization of gene expression was done relative to the housekeeping gene *RpL32* (*Rp49)*. For validation of RNA-Seq, fold expression change was calculated using the ΔΔCt method and data presented as a fold change to respective Tris-injected or non-infected controls. For all assays, relative gene expression values were converted as Log10 values. Ct cycle values and normalization for each RT–qPCR experiment are presented in Supplementary Table S3.

### Statistical analysis

Statistical analyses were performed using GraphPad Prism software (version 9.0.0) for MAC. Survival curves were compared using a Log-rank (Mantel-Cox) test. The effect of aging on virus load and ncRNA gene expression was analyzed by two-way ANOVA test followed by a Tukey’s multiple comparisons test. For validation of RNA-Seq gene expression by RT–qPCR, a two-tailed, parametric unpaired *t*-test was used for the comparison of two groups of samples. For all comparisons, *P *<* *0.05 was considered significant.

### Functional annotation analysis

We used the Database for Annotation, Visualization and Integrated Discovery (DAVID) 6.8 (Huang *et al.* 2009a, 2009b) to analyze enriched functional gene categories, including gene ontology (GO) and KEGG pathways for differentially regulated genes at least twofold. For both Gene ontology and KEGG pathway analysis, we used default options (in thresholds count was 2 and EASE score was set at 0.1). The cut-off non-corrected *P*-value to determine enriched GO categories and pathways was set at 0.1.

### Data availability

Raw sequencing reads generated during this project have been deposited with the National Center for Biotechnology Information Sequence Read Archive under BioProject PRJNA644593. File names corresponding to experimental samples are shown in Supplementary Table S1. The authors affirm that all data necessary for confirming the conclusions of the article are present within the article, figures, and tables, and in Supplementary material. Supplementary files including supplemental experimental procedures, figures, and tables have been deposited to the GSA figshare portal: https://doi.org/10.25387/g3.14348126. Supplementary Tables S3, S6, S8, and S9 have been submitted as Excel files while all other supplemental materials are in a PDF format.

## Results

### FHV infection leads to decreased survival but not increased virus load in aged *Drosophila*

To determine how age affects survival to infection with FHV, we individually injected 5- and 30-day-old wild-type (*Oregon R*) male or female flies with either Tris buffer (control) or FHV. In a set of 12 independent injection experiments, the survival of groups of 8–10 injected flies was recorded every 24 h. About 30-day-old flies showed significantly decreased survival in comparison to 5-day-old flies (*P* < 0.0001 for males and *P* = 0.0008 for females) (Figure 1, A and B, left panels). We observed that at 4 dpi both young and aged flies had similar survival rates (96.67% and 95.00% for males, respectively and 91.67% and 90.00% for females, respectively), while at 6 dpi, 91.67% of young males, 57.50% of aged males, 81.67% of young females, and 71.67% of aged females remained alive, respectively. At 7 dpi, we observed that 30.83% of the aged *vs* 77.5% of young males and 44.17% of the aged *vs* 60.17% of young females remained alive following infection with FHV (Figure 1, A and B).

**Figure 5:**
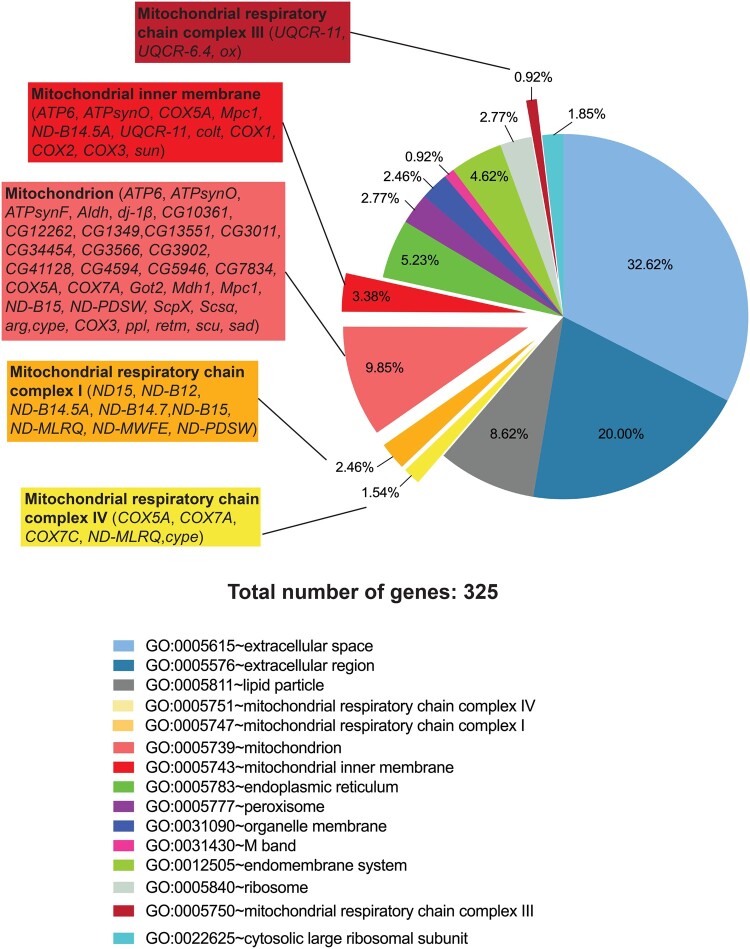
Gene ontology analysis for cellular component of genes specifically downregulated in aged, FHV-infected flies, reveal enrichment for mitochondria and mitochondrial respiratory chain complexes. GO analysis of at least twofold differentially regulated genes 48-h post-FHV infection in aged flies. All identified categories are shown.

Next, we hypothesized that the higher mortality in aged flies could result from increase in FHV load. To test this, we used quantitative reverse-transcription PCR (RT–qPCR) done using RNA isolated from groups of five male and female 5- and 30-day-old flies separately injected with either Tris or FHV. We measured virus load at 4, 5, and 6 dpi in both males and females as well as at 7 dpi in females. The additional 7-dpi time point for females was chosen because it represented the time post-infection (p.i.) when approximately half of the aged female flies died (55.83%). The approximately 50% death mark for FHV-infected aged males was at day 6 (42.5%). At all examined time points for both sexes, we observed comparable, non-significantly different (*P* > 0.05) levels of FHV RNA1 (*FHV1*) expression between young and aged flies (Figure 1, A and B, right panels). Interestingly, although survival curves of young flies overlapped between both sexes (Supplementary Figure S2A), in comparison to males, aged females showed slight, but significantly improved survival of FHV (*P* = 0.0025, Supplementary Figure S2B). At 4, 5, and 6 dpi, for both young and aged flies, females showed seemingly lower levels of *FHV1* expression in comparison to males. The mean ± SD *FHV1* Log10 values were as follows: at 4 dpi: 3.24 ± 0.04 for young females *vs* 3.61 ± 0.06 for young males and 3.25 ± 0.21 for aged females *vs* 3.59 ± 0.16 for aged males; at 5 dpi: 3.27 ± 0.23 for young females *vs* 3.42 ± 0.44 for young males and 3.03 ± 0.44 for aged females *vs* 3.42 ± 0.44 for aged males; and at 6 dpi: 3.09 ± 0.16 for young females *vs* 3.18 ± 0.11 for young males and 3.04 ± 0.32 for aged females *vs* 3.28 ± 0.15 for aged males. However, this difference in virus load between the two sexes at the examined time points after infection was not statistically significant (Supplementary Figure S2C).

In support of the data obtained for *Oregon R* flies, significant difference in survival between 5- and 30-day-old flies was observed for two other laboratory strains: *w^1118^* and *y^1^ w^67c23^* (*P* < 0.0001 for both mixed-sex cohorts of *w^1118^* and male *y^1^ w^67c23^* flies, Supplementary Figure S3, A and B, left panels). Additionally, for both genotypes, we observed comparable *FHV* loads between animals of the two age groups [*P* = 0.9903 for *y^1^ w^67c23^* males at 3 dpi measured by RT–qPCR and *P* = 0.7075 for *w^1118^* females at 4 dpi measured by TCID50 in circulating hemolymph (insect blood)] (Supplementary Figure S3, A and B, right panels).

Altogether, these results indicate that in comparison to younger individuals, aged *Drosophila* succumb faster to infection with FHV without an increase in FHV titers. This suggests that the aged organism is able to control viral pathogen burden at time points in the course of infection during which difference in survival between age groups is observed. Therefore, it is likely that an age-dependent impairment of disease tolerance accompanies the observed increase in mortality. Based on the observation that in comparison to aged females, aged males exhibit higher mortality after FHV infection, our results also point to a potential sexual dimorphism in the age-dependent survival of FHV infection.

### Aged *Drosophila* mount a robust transcriptional response following FHV infection

Both aging and virus infection lead to changes in the *Drosophila* transcriptome (Pletcher *et al.* 2002; Kemp *et al.* 2013; Chtarbanova *et al.* 2014). We hypothesized that aged flies infected with FHV mount a distinct transcriptional response in comparison to young flies, potentially accounting for the observed increase in mortality. To test this, we performed transcriptomics analysis using RNA sequencing (RNA-Seq) on 7- (young) and 25-day-old (aged) male *Oregon R Drosophila.* Triplicates of 15 flies injected with either Tris or FHV were collected for RNA extraction at 24 and 48 h following injection. This sex was chosen because aged males showed more pronounced effect on decrease in survival than females (Supplementary Figure S2B). The time points were chosen early in the infection process before differences in survival between age groups were detected. As an additional control, we used non-infected young and aged flies to control for the effects of aging alone in absence of infection. An average of 95.4% of each RNA-Seq library (Supplementary Table S4) aligned to the *D. melanogaster* genome (Supplementary Table S5).

To evaluate the overall similarity and differences between treatments, we used principal component analysis (PCA). We observed that both young and aged FHV samples displayed a composite signature of gene regulation fundamentally different from non-infected young, non-infected aged, or Tris-injected samples. PC1 explained 54% of the variance in gene expression and separated young and older FHV-infected hosts from non-infected controls. PC2 explained 20% of the variance in expression and further separated FHV-infected hosts 24- and 48 h p.i. Each of the three replicates grouped by treatment with the exception of the young Tris-injected flies 24- and 48 h p.i., which overlapped (Figure 2A).

Differential gene expression analysis comparing young and aged non-injected flies revealed that the process of aging itself significantly regulated (*P adj* < 0.05) at least twofold the expression of 1639 genes. In the absence of injury or infectious challenge, 424 genes were upregulated and 1215 genes were downregulated (Figure 2B and Supplementary Table S6). Intrathoracic Tris injection led to differential regulation of genes in both age groups. In young flies, in comparison to non-injected flies, a total of 231 (105 upregulated and 126 downregulated) and 505 (224 upregulated and 281 downregulated) genes were differentially expressed at 24- and 48 h p.i., respectively (Supplementary Figure S4A and Table S6). In aged flies, in comparison to non-injected flies, a total of 456 (361 upregulated and 95 downregulated) and 196 (136 upregulated and 60 downregulated) genes were differentially expressed at 24- and 48 h p.i., respectively (Supplementary Figure S4B and Table S6).

Differential gene expression analysis following FHV infection revealed that in comparison to Tris-injected controls, more genes were significantly regulated (*P adj* < 0.05) at least twofold at 48 h p.i. in comparison to 24 h p.i. in both age groups. More genes were differentially changed in aged FHV-infected flies in comparison to young flies for both time points. Overall, in young flies, the expression of 505 genes was differentially changed 24 h p.i. *vs* 1168 genes 48 h p.i. In aged flies, we observed differential regulation of 816 genes at 24 h p.i. and 2625 genes at 48 h p.i. (Figure 2B and Supplementary Table S6).

We validated the RNA-Seq data for aging and the 48-h post-FHV infection time point in both young and aged flies using specific primers and RT–qPCR analysis for four genes per experimental condition. We confirmed that in aging flies *Cpr67Fb* and *CG15199* were upregulated and *Acp54A1* and *Lman III* were downregulated. In young *Drosophila*, 48 h after FHV infection, *Upd3* and *Socs36E* were upregulated and *Rfabg* and *Diedel 3* were downregulated in comparison to Tris-injected controls. In aged *Drosophila*, 48 h after FHV infection, *Or85a* and *Upd3* were upregulated and *IM14* and *GNBP-Like 3* in comparison to Tris-injected controls (Supplementary Figure S5). The *Upd3* gene, which we find upregulated in both young and aged flies, encodes for a protein that together with Upd1 and Upd2 belongs to the Unpaired family of cytokine-like proteins, and which is a ligand for the receptor Domeless (Dome). Upd binding to Dome leads to activation of the evolutionarily conserved JAK/STAT pathway, for which roles in *Drosophila* development, stress response, and antiviral immunity have been previously reported (Schneider and Imler 2021). Upd3 upregulation could result from cell damage caused by viral infection, and its regulation to a greater extent in the aged fly could possibly reflect a more extensive tissue damage caused by FHV infection in older flies.

Among the genes differentially regulated during aging, we observed a very small overlap with genes regulated by infection at either young or older age, 24- or 48 h p.i. (1.4% and 2.5%, respectively) (Figure 2C). At 24 h p.i., ∼50% of upregulated genes and 57% of downregulated genes in young flies overlapped with genes upregulated in aged, FHV-infected flies. At 48 h p.i. in young flies, 93% of upregulated genes overlapped with up-regulated genes in FHV-infected aged flies and 57% of downregulated genes overlapped between the two age groups (Figure 2C).

Altogether, these results indicate that in comparison to Tris-injected controls, aged male flies mount a larger transcriptional response following FHV infection than younger flies. This signature appears different from the transcriptional changes taking place during the aging process alone as shown by the minimal overlap of differentially regulated genes between these conditions. The fact that most of commonly regulated genes between young and aged FHV-infected flies were found to overlap as a function of time (86% of up- and 87% of downregulated genes, Supplementary Figure S6) supports of the hypothesis that the age-dependent defect in disease tolerance is unlikely to result from the regulation of these genes. Rather, our data suggest that impaired tolerance in aged flies could be due to differential regulation of the genes that are uniquely expressed in infected young flies, uniquely expressed in infected aged flies, or a combination of both.

### FHV infection triggers transcriptional changes in similar and different biological processes in young and aged *Drosophila*

To visualize biological processes (BPs) regulated by aging, injury, and FHV infection in young and aged flies, we performed gene ontology (GO) analysis using the Database for Annotation, Visualization and Integrated Discovery (DAVID) (Huang *et al.* 2009a, 2009b). The number of genes with Flybase ID (FBgn number) without a matching DAVID ID is listed in Supplementary Table S7. We note that most differentially regulated genes with a DAVID ID were labeled as “Others” (Supplementary Figure S7). For instance, 76% of differentially regulated genes for the Aging group did not match a specific BP. For Young Tris24h, Young Tris48h, Aged Tris24h, Aged Tris48h, Young FHV24h, Young FHV48h, Aged FHV24h, and Aged FHV48h, these percentages are 56%, 77%, 55%, 56%, 53%, 59%, 60%, and 59%, respectively (Supplementary Figure S7).

Our GO analysis revealed a complex signature. For instance, aging alone led to changes in expression of genes belonging to 57 BPs. In response to the injury alone, in young flies, we identified 49 and 38 BPs at 24- and 48-h post-Tris-injection, respectively. In aged flies, Tris-injection led to changes in gene expression belonging to 52 and 28 BPs at 24 and 48 h, respectively. In comparison to Tris-injected flies, in young flies FHV infection led to changes in 96 and 81 BPs at 24- and 48 h p.i., respectively. And finally, in comparison to aged Tris-injected flies, aged flies infected with FHV differentially regulated the expression of genes belonging to 80 and 135 BPs at 24- and 48 h p.i., respectively (Supplementary Table S8).

In Figure 3A, is represented the number of BPs GO categories, which are specific to, or overlap between, the five experimental conditions Aging, Young FHV 24 h, Young FHV 48 h, Aged FHV 24 h, and Aged FHV 48 h. This comparison was done in order to identify BPs, that could possibly account for the impaired disease tolerance phenotype observed in aged, FHV-infected flies.

We found that five GO BPs (“defense response,” “response to bacterium,” “antibacterial humoral response,” “defense response to Gram-positive bacterium,” and “oxidation–reduction”) overlapped between all five experimental conditions. “Mannose metabolic process” and “protein refolding” were in common between Aging and Aged FHV24h groups and “sperm storage” between Aging and Aged FHV48h groups. Processes identified in common between the Aging group and young and aged FHV-infected flies were “circadian rhythm,” “multicellular organism reproduction,” and “proteolysis” (Figure 3B and Supplementary Table S8). In *Drosophila*, aging leads to both, deregulation of organismal reproduction (Tatar 2010) and innate immunity (Pletcher *et al.* 2002; Zerofsky *et al.* 2005; Kounatidis *et al.* 2017). In flies, it is also well established that physiological trade-offs exist between immune activation and reproductive capacity (Zerofsky *et al.* 2005), potentially accounting for the differential regulation of genes involved in organismal reproduction after FHV infection in both, young and aged flies. We note that distinct effects on seminal protein expression are observed in aging mated males in comparison to aging virgin males (Sepil *et al.* 2020). How the mating status of the aged fly impacts the transcriptomic changes related to organismal reproduction in response to FHV infection could be addressed in future experiments. Among the 46 BPs specific to Aging, we find genes belonging to “metabolic process” and “spermatogenesis” GO categories (Figure 3 and Supplementary Table S8). This aligns with previous studies showing that aging impacts male germline stem cells and leads to decrease in spermatogenesis (Boyle *et al.* 2007), which is mating-independent (Sepil *et al.* 2020), and with previous observations that the aging process leads to differential regulation of genes involved in *Drosophila* metabolism (Pletcher *et al.* 2002).

In comparison to Tris-injected controls, at 24-h post-FHV infection, we identified more BPs in young flies than in aged animals (96 *vs* 80, respectively). At 48 h p.i., we found an opposite trend with 81 and 135 BPs in young and aged flies, respectively. At 24 h p.i., five BPs overlapped between the two age groups, while 23 BPs overlapped between young and aged flies at 48 h p.i. (Figure 3A and Supplementary Table S8). About 70 and 26 BPs were specific to Young FHV24h and Aged FHV24h, respectively, while 20 and 63 BPs were specific to the Young FHV48h and Aged FHV48h groups, respectively (Figure 3, A and B and Supplementary Table S8).

In both young and aged flies, FHV infection led to differential regulation of genes involved in processes associated with the nervous system. Clustering analysis identified one module of “neurogenesis” genes that were strongly upregulated in the Aged FHV48h group and regulated to a lesser extent in Young FHV48h and Aged FHV24h groups (Figure 3 and Supplementary Figure S8A and Table S8). For instance, among genes belonging to this GO category at 48 h p.i., the gene *midlife crisis* (*mdlc*), which is required for neuroblast proliferation and neuronal differentiation in *Drosophila* (Carney *et al.* 2013), was upregulated to a greater extent in aged, FHV-infected flies. *Ankyrin repeat and LEM domain containing 2* (*Ankle2*), the *Drosophila* ortholog of human ANKLE2, which is a target of the ZIKA virus NS4 protein (Shah *et al.* 2018), also showed stronger upregulation in aged FHV-infected flies (Supplementary Figure S8B and Table S8). Other BPs linked to the nervous system development and function for which genes were enriched in young and aged FHV-infected groups were “lateral inhibition,” “sleep,” and “ventral cord development” (Figure 3 and Supplementary Table S8). The significance of this regulation is not known as FHV has not been previously demonstrated to target the nervous system, but rather the *Drosophila* heart, fat body, trachea, and ovarian egg chamber (Galiana-Arnoux *et al.* 2006; Eleftherianos *et al.* 2011; Thomson *et al.* 2012). Among other BPs identified in common between young and aged FHV-infected flies, we find “innate immune response,” “protein folding,” “rRNA processing,” and “response to heat.” In *Drosophila*, the heat shock response plays an antiviral role against the RNA viruses DCV and Cricket Paralysis Virus (CrPV), as well as against the DNA Invertebrate Iridescent Virus 6 (IIV-6) (Merkling *et al.* 2015b). Indeed, several HSPs belonging to the BP “response to heat” were upregulated in both young and aged FHV-infected flies (Supplementary Tables S6 and S8). This suggests that following FHV infection, this branch of antiviral immunity is preserved in aged flies.

Interestingly, genes belonging to additional categories associated with nervous system’s function such as “neuromuscular synaptic transmission,” “transmembrane transport,” and “neurotransmitter secretion” were specifically found in the Young FHV24h group. On the other hand, among processes specific to Aged FHV24h, we found “autophagic cell death” and “regulation of autophagy” (Figure 3 and Supplementary Table S8). Autophagy is an evolutionarily conserved cellular process previously reported to play a role in antiviral defenses in *Drosophila* (Lamiable and Imler 2014). Among processes specifically enriched 48 h p.i. in aged FHV-infected flies, we found “regulation of transcription,” “DNA-templated,” “transmembrane receptor protein tyrosine kinase signaling pathway,” and “protein ubiquitination” in young flies and “phagocytosis,” “programmed cell death,” and “peptidoglycan recognition protein signaling pathway.” The latter category contained multiple genes encoding for components of the *Drosophila* IMD pathway. Finally, among the processes specifically regulated in aged flies at both 24- and 48 h p.i., we found “apoptotic process,” “determination of adult lifespan,” and “chromatin remodeling” (Figure 3 and Supplementary Table S8).

Overall, these results indicate that despite a large number of “other” genes, genes belonging to identifiable common and distinct categories of BPs are regulated by aging and FHV infection of young and aged flies. Although our results identify specific categories of BPs for each experimental group (Figure 3 and Supplementary Table S8), at this stage, we are not able to determine whether the age-associated impairment of disease tolerance depends on the regulation of genes that are specifically regulated in young or/and aged flies.

### Profiles of innate immunity gene expression are distinct between injury and FHV infection in young and aged flies

We found genes belonging to the “innate immune response” BP category to be differentially regulated across Tris-injected and FHV-infected groups in both young and aged flies at the 24 and 48 h time points (Supplementary Table S8). We sought to determine whether these responses differed between the Tris and FHV conditions. To that end, we performed clustering analysis for this GO category. Results from this analysis revealed an increase in the expression pattern of innate immune response-related genes in absence of injury or infectious challenge (Aging group). Consistent with previous reports, we observed significantly increased expression of several AMP and IM genes (*CecA1*, *Def*, *IM3*, *Drs*, *IM2*, *IM1*, *IM4, IM14*, and *IM33*) as well as *GNBP-like 3* in aging flies (Figure 4A and Supplementary Figure S9 and Table S6).

**Figure 6:**
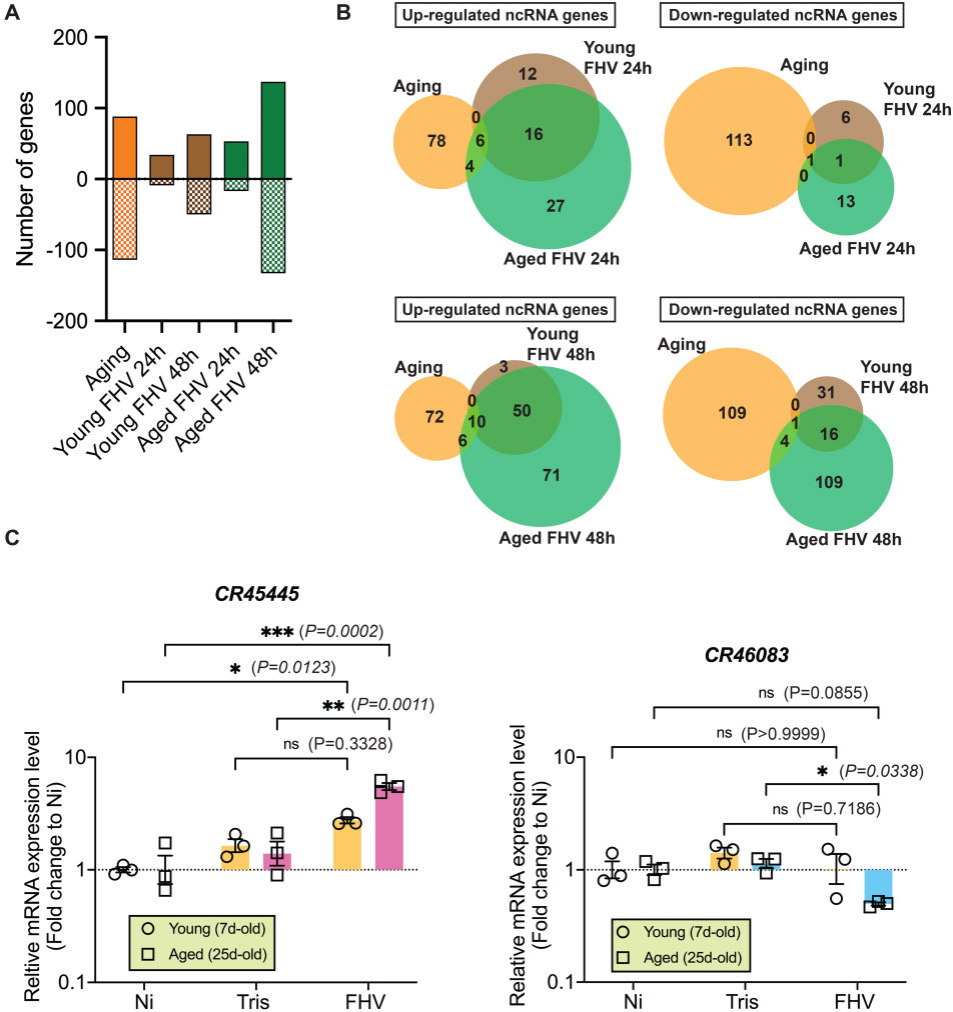
ncRNAs are differentially regulated by aging and FHV infection. (A) Comparison of the number of differentially regulated genes for ncRNAs at least twofold in all conditions. Positive values represent upregulated genes and negative values represent downregulated genes. (B) Venn diagrams showing overlaps between differentially regulated ncRNA genes for selected experimental conditions. (C) RT–qPCR-based gene expression analysis of asRNA *CR45445* and lncRNA *CR46083* 48 h p.i. Graphs represent mean ± SEM from three independent experiments and each symbol represents a group of 15 flies. Statistics are based on two-way ANOVA followed by Tukey post-test to correct for multiple comparisons. ****P < 0.0001, **P < 0.001, *P < 0.05, ns = non-significant.

In comparison with non-injected flies, in young and aged flies Tris injection affected the expression of several *Turandot* (*Tot*), IM and AMP genes both at 24- and 48 h p.i. (Figure 4A and Supplementary Table S6). This response was stronger at 24-h post-Tris-injection in both age groups and although it remained regulated to a greater extent in older animals, it decreased at the 48 h time point (Figure 4A and Supplementary Table S6). About 28 upregulated genes overlapped across the Young Tris24h, Young Tris48h, Aged Tris24h, and Aged Tris48h groups (Supplementary Figure S4B). Among these genes, we find several encoding for AMPs, including *Attacin-C*, *Diptericin*, *Diptericin B*, *Metchnikowin*, *Drosocin* as well as *IM4* (Supplementary Figure S4C). These results indicate that in response to Tris injection both young and aged animals respond by upregulating innate immunity genes.

Our analysis identified similar patterns of differential gene expression between young and aged, FHV-infected flies; regulation, which was to a greater extent in aged, FHV-infected flies (Figure 4A). Interestingly, in both young and aged animals, in comparison to Tris-injected controls, FHV infection led to strong downregulation of most AMP and IM genes, despite a robust upregulation of the mRNA encoding the NF-κB factor Relish (Figure 4A and Supplementary Table S6). In aged FHV-infected flies, we observed marked upregulation of IMD pathway components *PGRP-LE*, *imd*, *key* (*IKKγ*), and *AttD*. This upregulation was to a greater extent in the Aged FHV48h group (Figure 4A and Supplementary Figure S9). In comparison to aging and young FHV-infected *Drosophila*, we found *dSTING*, whose product acts upstream of Relish to protect flies against infection with DCV and CrPV (Goto *et al.* 2018), to be strongly upregulated in aged FHV-infected flies (Supplementary Figure S9).

Altogether, these results indicate that aging, injury, and virus infection lead to changes in transcriptional signatures for innate immunity genes. However, the signature and extent of regulation differ between conditions. Additionally, in comparison to Tris-injected controls, aged flies carry out an overall stronger response to FHV than younger flies and regulate expression of more components of the IMD pathway. Because overactivation of the IMD pathway exerts detrimental effects on *Drosophila* tissues and leads to premature death (Cao *et al.* 2013; Kounatidis *et al.* 2017), our results also suggest that the specific upregulation of components of this pathway could be responsible for impaired tolerance and decreased survival in aged FHV-infected flies.

### Strong apoptotic gene expression signature in aged *Drosophila* following FHV infection

“Apoptotic process” was among the GO categories represented specifically in aged FHV-infected flies (Figure 3 and Supplementary Table S8). Apoptosis is a form of programmed cell death, which has previously been linked to FHV infection. For instance, p53-dependent early induction of pro-apoptotic genes has been implicated as a protective mechanism against FHV infection in *Drosophila* (Liu *et al.* 2013). Additionally, infection with FHV of *Drosophila* cells in culture leads to induction of apoptosis, which is dependent on the effector caspase DrICE, the initiator caspase Dronc, and its cofactor Dark (Settles and Friesen 2008). Consistent with this, clustering analysis for this GO category showed that FHV infection in both age groups leads to transcriptional changes in expression of several genes involved in the apoptotic process including *p53*, *Dronc*, and *Dark* (Figure 4B). Markedly, the upregulation of genes belonging to this BP was to a greater extent in the Aged FHV48h group. In cell culture, over the course of FHV infection, protein levels of the *Drosophila* inhibitor of apoptosis (Diap-1) are progressively depleted as a possible result of host cell translational shut down (Settles and Friesen 2008). In our RNA-Seq data, we find that *diap-1* mRNA increased post-infection and to higher levels in aged flies in comparison to young adults (Figure 4B). This change could potentially represent a compensatory increase in *diap-1* mRNA as a result of the rapid depletion of the protein.

Collectively, these results are in agreement with previous findings that FHV infection leads to apoptotic cell death. The stronger extent of upregulation observed in aged, FHV-infected flies suggest that either more rapid or widespread activation of cell death takes place in the aged, FHV-infected organism.

### Genes specifically downregulated in aged flies following FHV infection are enriched for metabolic pathways and mitochondria

We observed a larger number of genes to be specifically regulated 48 h p.i. in aged flies in comparison to younger adults after FHV infection (upregulated genes: 822 in Aged FHV48h *vs* 48 in Young FHV48h; downregulated genes: 778 in Aged FHV48h *vs* 149 in Young FHV48h, Figure 2C). We performed both Gene ontology and pathway enrichment analysis on the genes specifically up- and downregulated in the Aged FHV48h group (822 upregulated and 778 downregulated genes, respectively; Figure 2C) using the DAVID database. Our GO analysis of upregulated genes for BPs identified 86 BP categories, while GO analysis of downregulated genes identified 43 BPs (Supplementary Table S9). Among Kyoto Encyclopedia of Genes and Genomes (KEGG)-mapped pathways, we found six to be specifically enriched among upregulated genes, including “purine metabolism,” “pyrimidine metabolism,” and “RNA polymerase” (Supplementary Figure S10 and Table S9). This likely reflects the higher transcriptional rates observed in aged animals in comparison with younger adults following FHV infection. Among KEGG-mapped pathways for downregulated genes, we found 29, which included “metabolic pathways,” “biosynthesis of antibiotics,” and “oxidative phosphorylation” (Supplementary Figure S10 and Table S9). GO analysis for cellular component (CC) among upregulated genes, identified 29 categories, including “nucleus,” “nucleolus,” and “cytoplasm” (Supplementary Table S9). For downregulated genes, we identified 15 CC categories, including “mitochondrion” and “mitochondrial respiratory chain complex” I, III, and IV (Figure 5). Mitochondrial respiratory chain complex, also referred to as mitochondrial electron transport chain (ETC) complexes represent a series of four protein complexes (I–IV) distributed along the inner mitochondrial membrane, where they function to pump protons from the mitochondrial matrix into the intermembrane space. ETC complexes are coupled to Complex V (the ATP synthase), which helps the production of ATP (Burke 2017). The observed downregulation of these genes could reflect a virus-induced defect of the mitochondrial respiratory chain affecting ATP levels, specifically in the infected aged organism. This could be the direct result of FHV replication on mitochondrial membranes, or, an indirect effect of FHV-induced apoptotic cell death.

### Non-coding RNAs are differentially regulated by aging and FHV infection

We took a closer look at the differentially regulated genes, which were labeled as “other” in our GO analysis (Supplementary Figure S7). We observed that most of these genes are uncharacterized (categorized as candidate genes, or CG); several are ncRNA; and others have previously described function but do not fit a specific DAVID GO category. Among ncRNAs, long non-coding RNAs (lncRNAs) correspond to a class of transcripts, which are at least 200 nt long and lack a significant open reading frame (reviewed in Sun and Kraus 2015). Most lncRNAs are polyadenylated and can be reliably identified in our RNA-Seq workflow, in which an oligo-dT-based enrichment of poly-A-containing transcripts was used. A class of lncRNAs corresponds to antisense (as) RNAs, which are natural antisense transcripts (NATs) that overlap with protein-coding *loci* in the antisense direction.

We compared the number of ncRNAs differentially regulated at least twofold in our RNA-Seq dataset (Supplementary Table S6) and observed changes in expression of higher number of ncRNAs genes in aged FHV-infected than in young FHV-infected flies (68 *vs* 42 genes 24 h p.i. and 267 *vs* 111 genes 48 h p.i.). Aging itself regulated the expression of 202 ncRNA genes (Figure 6, A and B). As observed for the total number of transcripts (Figure 2C), ncRNAs, which were regulated by infection shared minimal overlap with aging. For upregulated ncRNA genes, only 6.8% and 11.36% of genes regulated by aging overlapped between all three experimental conditions at 24- and 48 h p.i., respectively. For downregulated ncRNA genes, these percentages were 0.88% and 3.5% at 24- and 48 h p.i., respectively (Figure 6B). Among ncRNAs, we identified the largest proportion to correspond to lncRNAs. For all experimental groups, we also found asRNAs and small nucleolar RNAs (snoRNAs). In young FHV-infected flies, a small percentage of ncRNAs corresponded to stable intronic sequence RNAs (sisRNAs). Specifically, in aged, FHV-infected flies, we found differential regulation of ncRNAs that belong to small nuclear (snRNAs) and small non-messenger RNAs (snmRNAs) (Supplementary Figure S11).

We compared the expression of *CR45445* (an asRNA) and *CR46083* (an lncRNA) genes 48 h p.i. by RT–qPCR. Consistent with the RNA-Seq data, we observed significant increase in *CR45445* and significant decrease in *CR46083* expression in comparison to Tris-injected controls in aged, but not young flies (Figure 6C). Together, these results indicate that both, aging and FHV infection affect the expression of genes encoding different categories of ncRNAs, and that specific ncRNAs are regulated in the aged organism after FHV infection.

## Discussion

We used the highly tractable genetic model *D.* *melanogaster* to investigate the response of the aged organism following infection with the RNA(+) virus FHV. We found that 30-day-old *Oregon-R* flies died faster than younger flies to FHV infection and that older, but not younger males were more sensitive than females. Although our results raise the interesting question of whether survival of virus infection in the aged organism represents a sexually dimorphic trait, we cannot exclude the possibility that this is due to genetic background-specific effects. Although FHV load appeared consistently lower in both young and aged females than in males, this difference was not significant. Future studies examining survival outcomes, virus load, and gene expression in males and females of other *D.* *melanogaster* genetic backgrounds (*e.g.*, control lines such as *Canton S*, *w^1118^*, and *y w*) could provide additional information about the extent of our findings. It is increasingly recognized that sexual dimorphism in immune function exists in *Drosophila* although the precise mechanisms underlying these age-dependent dimorphic differences are poorly understood (reviewed in Belmonte *et al.* 2019). Therefore, more work is needed to elucidate this important aspect of antiviral immunity.

Both resistance and tolerance are components of host immunity (Ayres and Schneider 2012; Martins *et al.* 2019). Antiviral RNAi is the main resistance mechanism that defends *Drosophila* against a broad range of RNA and DNA viruses, including FHV (Kemp *et al.* 2013). RNAi pathway mutants such as *dicer-2* mutants, are more sensitive to FHV infection and mortality in *dicer-2* mutants is accompanied by higher virus loads (Galiana-Arnoux *et al.* 2006). In this study, we find comparable FHV titers between young and aged flies in both whole bodies and circulating hemolymph. This suggests that aging likely affects tolerance mechanisms instead of resistance mechanisms. Earlier studies demonstrated that older *Drosophila* exhibit higher mortality following infection with *Escherichia* *coli*, but were able to clear bacteria at similar rates as young flies (Ramsden *et al.* 2008) despite age-associated decline in macrophage function (Mackenzie *et al.* 2011; Horn *et al.* 2014). Both humoral (*e.g.*, induction and secretion of AMPs) and cellular (*e.g.*, phagocytosis) responses are required for bacterial clearance. It was proposed that the increase in AMP expression that accompanies normal aging could possibly compensate for decreased phagocyte function and account for the absence of an increase in bacterial load. Thus, the increased mortality following bacterial infection likely relies on age-dependent defects in tolerance (Ramsden *et al.* 2008). In our transcriptomic analysis, we do not find noticeable transcriptional changes in gene expression of RNAi pathway components with aging, at least when flies are aged up to 25 days. This indirectly supports the hypothesis that antiviral RNAi is not functionally impaired in the aged fly. However, additional studies including small RNA sequencing during aging to compare the abundance of siRNAs against the FHV genome, are needed to determine whether this is the case.

We cannot entirely rule out the possibility that aging impacts resistance mechanisms in a tissue-specific way, differences in which cannot necessarily be detected by measuring virus load in whole flies. It therefore would be very informative to perform additional studies to determine whether FHV differentially targets tissues at different ages and whether FHV load differs among tissues as a function of age. For instance, it is appreciated that aging affects gene expression differently in different tissues and in mammalian models differentially expressed genes in a given tissue are often not genes specific to this tissue (Rodwell *et al.* 2004). In *Drosophila*, a temporal and spatial transcriptional study of aging done on seven different tissues identified that <10% of differentially expressed genes in each tissue were in common with any other tissue (Zhan *et al.* 2007). It is therefore possible that host factors required for virus tissue tropism at younger age (*e.g.*, in the heart and fat body; Eleftherianos *et al.* 2011) become expressed in a different tissue in the aged host leading to shift in virus tropism accompanied by increased mortality even in the absence of higher virus titers. The aged *Drosophila–*FHV system could therefore represent an excellent model to address these questions and further examine how the aged organism is affected in the course of virus infection.

One striking finding of this study is that aged flies infected with FHV mount a more robust transcriptional response than younger flies in the early time points of infection. The fact that at 48 h after FHV infection we find an overlap between 93% of upregulated genes and 57% of downregulated genes in young flies with genes regulated in aged flies, suggests that most of the transcriptional response to FHV is maintained as a function of age at the examined time points. However, aged flies show extensive regulation of additional genes. One possibility was that these additional genes are related to the process of aging itself. We show, however, that the overlap between the transcriptional profiles of aging, non-infected flies and aged, FHV-infected flies is minimal. The observed difference can potentially account for the changes in tolerance with age. Approximately three times more genes are downregulated than upregulated in aging flies in absence of viral infection. In aged, FHV-infected flies, we observe the opposite: a higher number of upregulated than downregulated genes for both time points examined. Thus, compared to younger adults, the aged fly mounts somehow a distinct response following FHV infection that is the consequence of the response to the virus rather than the process of aging itself. More studies are needed to further dissect the immunopathological mechanisms triggered in response to FHV in the aged host. It would be particularly interesting to establish whether specific regulatory mechanisms that dampen host antiviral responses remain activated at later time points of infection and therefore associate with tolerance impairment. We observed that in aged male flies 6 days after FHV infection, the expression of *Attacin C*, which is a downstream target of the IMD pathway, remained elevated approximately fivefold in comparison to young, FHV-infected flies. This difference, however, was not statistically significant (Supplementary Figure S12). Whether prolonged IMD pathway activation results in immunopathology in the context of FHV infection in the aged host should remain the focus of future research. Additional transcriptomic analysis at later time points of infection, during which significant differences in survival between age groups are observed and also warranted. Such analysis could help to draw a more complete picture about differential regulation of genes and virus-induced immunopathology in insects, which remains a largely understudied field (Gonzalez-Gonzalez and Wayne 2020).

It also remains unclear what factors contribute to the stronger transcriptional signature seen in aged FHV-infected flies. One hypothesis is that this possibly results from regulation by ncRNAs, including lncRNAs, which can play a role in gene expression regulation (Sun and Kraus 2015). Indeed, we find several lncRNAs regulated by infection specifically in aged, FHV-infected flies. Because lncRNAs are able to interact with DNA, RNA, and proteins, these molecules could influence gene expression at multiple levels, including transcription, RNA-processing, translation, and post-translation (He *et al.* 2018). lncRNAs are increasingly recognized to participate in the regulation of both aging and immunity (Atianand *et al.* 2017; He *et al.* 2018). In mammalian models, lncRNAs have been shown to affect multiple molecular traits of aging, including telomere length, cell proliferation, and proteostasis (Grammatikakis *et al.* 2014; He *et al.* 2018). Additionally, lncRNAs play various roles in mammalian innate and adaptive immunity, including differentiation of immune cell lineages, regulation of cytokines gene expression and inflammatory responses, as well as lymphocyte development and activation (Atianand *et al.* 2017). A recent study in *Drosophila* identified the virus suppressor of RNAi (VSR)-interacting lncRNA (VINR): an antiviral lncRNA that becomes upregulated after infection with DCV, to activate a Cactin-mediated non-canonical innate immune defense mechanism (Zhang *et al.* 2020). In this study, which used young 4- to 6-day-old flies, the authors reported that it was the DCV 1A, but not the FHV B2 VSR, which mediated VINR upregulation, leading to increased AMP expression via the Cactin-Deaf1 pathway (Zhang *et al.* 2020). Our experimental system of aged *Drosophila*–FHV interactions could be used in future experiments to address the question of whether specific lncRNAs regulate the larger transcriptional response to FHV seen in aged flies as well as to examine lncRNA function in response to infection as a function of age.

Our study finds that the gene encoding the NF-kB transcription factor Relish as well as some of the additional core components of the IMD pathway such as the adaptor protein Imd and the IKK complex component Key (IKKγ) are upregulated following FHV infection in aged flies 48 h p.i. One possibility is that this upregulation is due to FHV-induced leakage of the *Drosophila* gut microbiota. Indeed, the fly microbiota seems to partially contribute to the age-dependent increase in NF-kB-mediated gene expression in absence of overt infections (Kounatidis *et al.* 2017). Comparison of NF-kB signaling gene expression between young and aged axenic FHV-infected flies could help to address this question. Interestingly, however, we find several AMP and IM genes that normally are upregulated in NF-kB-dependent way upon bacterial and fungal infections including the fly microbiota (Broderick *et al.* 2014; Kounatidis *et al.* 2017), to be downregulated following FHV infection at both 24- and 48 h p.i., even in aged flies. The role of NF-kB pathways in *Drosophila* antiviral immunity is complex and still not fully elucidated; however, the pattern of expression that we see here in comparison to Tris-injected controls aligns with previous findings, where infection of S2* cells with the DNA virus IIV-6 leads to downregulation of AMP genes, despite intact cleavage and nuclear translocation of Relish (West *et al.* 2019). In the same study the authors report that although the expression of several AMP genes increases at 12-h post-IIV-6 infection *in vivo*, in comparison to PBS-injected controls, it returns to baseline or below baseline at 24- and 48 h p.i. Repression of AMP gene expression following IIV-6 infection appears downstream of Relish and likely occurs at the level of Relish binding to the AMP gene promoter or at the level of transcriptional activation (West *et al.* 2019). Whether in the case of FHV infection in aged flies the strong AMP and IM gene repression is mediated by similar mechanisms will remain a focus of future research.

Our results indicate that aged flies strongly upregulate *dSTING* expression in response to FHV 48 h p.i. In young flies, dSTING does not appear to play a protective role against FHV, as *dSTING* null mutants show similar, if not slightly better, survival to FHV in comparison to controls (Goto *et al.* 2018). It would be therefore interesting to examine the effect of the *dSTING* mutation in aged flies in response to FHV infection and determine whether this factor plays an antiviral role against this virus specifically in older flies. It may also be that in response to FHV, dSTING in aged flies plays a pro-death, rather than pro-survival role. In addition to its essential role in interferon production, STING signaling in mammals plays a role in the activation of programmed cell death, including Caspase-9 and Caspase-3-mediated apoptosis, although the exact mechanisms are not well understood (reviewed in Maelfait *et al.* 2020). Thus, it is possible that in response to FHV, dSTING mediates the strong apoptotic signature, that could be associated with the more rapid death observed in aged flies. Future analysis of *dSTING* function in older flies in response to FHV could for instance reveal novel information about evolutionary conservation of dSTING-mediated apoptotic signaling. Additionally, because of increased apoptotic gene deregulation and the fact that phagocytic function decreases with age, future experiments should be also aimed at examining whether defective apoptotic corpse clearance is associated with the impaired disease tolerance and higher mortality of older flies following FHV infection.

Our transcriptomic analyses reveal that as FHV infection progresses in aged flies, genes associated with mitochondrial respiratory chain become downregulated. This could be due to the direct interaction of FHV with the mitochondrion. It is well established both in cell culture and *in vivo* that FHV RNAs replicate on complexes at the outer mitochondrial membrane and induce the formation of characteristic spherule-like structures (Kopek *et al.* 2007; Eleftherianos *et al.* 2011). This characteristic spherule formation has been demonstrated in cardiac myocytes of young flies (Eleftherianos *et al.* 2011), however, we are lacking information about FHV replication dynamics on mitochondrial membranes of aged flies. Additionally, if FHV directly induced downregulation of ETC genes, this would likely be due to a specific interaction of the virus and the aged mitochondrion, as the strong twofold change in gene expression is observed in older flies. Aging is known to impact mitochondrial morphology and *Drosophila* mitochondria isolated from aged whole flies show altered cristae organization and inner mitochondrial membrane breakage (Brandt *et al.* 2017). Therefore, examining mitochondrial ultrastructure in aged, FHV-infected flies could reveal novel insights about the interaction of the virus and host cells. Among differentially expressed genes, we notice that several transcripts of genes encoded by the mitochondrial genome (Supplementary Table S6) are detected in the Aged FHV samples, especially at the early, 24 h time point, suggesting a link to apoptosis. One possible scenario is that p53-mediated apoptotic cell death is activated early in response to FHV leading mitochondria to become leaky and to release transcripts of genes that are encoded by the mitochondrial genome. Induction of pro-apoptotic gene expression within the first hours immediately following FHV infection of adult flies is an important mechanism that limits virus replication (Liu *et al.* 2013). It is important to examine the dynamics of this response in young and aged flies to determine whether any differences in this very early response are present between the two age groups. FHV-triggered apoptosis in the aged fly can also account for the downregulation of genes involved in the ETC and generation of ATP. As a consequence, it is possible that the bioenergetic profile of the cell is reduced and mitochondrial respiration halted post-infection in aged flies. Because programmed cell death and ATP production are increasingly considered closely linked aspects of mitochondrial function (Burke 2017), it will be important for future studies to determine whether FHV triggers apoptosis-dependent changes in cellular bioenergetics and how this relates to the more rapid death of the aged, FHV-infected organism.

In conclusion, in this study, we addressed for the first time how aged *Drosophila* respond to infection with the plus-strand RNA virus FHV and provide a detailed transcriptional comparison of the responses between young and aged flies at two time points following infection. With the advantages that *Drosophila* offer to investigate gene function, this study sets up the stage for future investigations about the mechanisms that underlie aged host–virus interactions using not only FHV, but also other viruses. For instance, although DCV triggers distinct pathophysiological events and transcriptional changes in comparison with FHV (Kemp *et al.* 2013; Chtarbanova *et al.* 2014), it also leads to the more rapid death of older flies (Eleftherianos *et al.* 2011). It would be very interesting to explore the age-dependent response to DCV infection, as this could lead to the discovery of additional mechanisms that help the aged organism survive virus infection.
